# Deficits in Tactile Learning in a Mouse Model of Fragile X Syndrome

**DOI:** 10.1371/journal.pone.0109116

**Published:** 2014-10-08

**Authors:** Megan T. Arnett, David H. Herman, Aaron W. McGee

**Affiliations:** 1 Developmental Neuroscience Program, Saban Research Institute, Children’s Hospital Los Angeles, Department of Pediatrics, Keck School of Medicine, University of Southern California, Los Angeles, California, United States of America; 2 Section of Neurobiology, Department of Biological Sciences, University of Southern California, Los Angeles, California, United States of America; CNRS UMR7275, France

## Abstract

The fragile X mental retardation 1 mutant mouse (*Fmr1* KO) recapitulates several of the neurologic deficits associated with Fragile X syndrome (FXS). As tactile hypersensitivity is a hallmark of FXS, we examined the sensory representation of individual whiskers in somatosensory barrel cortex of *Fmr1* KO and wild-type (WT) mice and compared their performance in a whisker-dependent learning paradigm, the gap cross assay. *Fmr1* KO mice exhibited elevated responses to stimulation of individual whiskers as measured by optical imaging of intrinsic signals. In the gap cross task, initial performance of *Fmr1* KO mice was indistinguishable from WT controls. However, while WT mice improved significantly with experience at all gap distances, *Fmr1* KO mice displayed significant and specific deficits in improvement at longer distances which rely solely on tactile information from whiskers. Thus, *Fmr1* KO mice possess altered cortical responses to sensory input that correlates with a deficit in tactile learning.

## Introduction

Fragile X Syndrome (FXS) is a leading inheritable cause of mental impairment, affecting approximately 1∶4000 males and 1∶8000 females in the United States [1]. FXS arises from a loss-of-function in the FMR1 gene that encodes the Fragile X Mental Retardation protein (fmrp), an RNA binding protein [2], [3]. Although the symptoms of FXS patients vary in severity and expression, characteristic phenotypes include reduced intellectual abilities, hyperactivity, increased seizure susceptibility, and impaired visuo-spatial processing [4]. Hypersensitivity to sensory stimuli, including tactile defensiveness, are also principal symptoms of FXS [5], [6]. At present, there is no known cure for FXS or treatment than reverses the collective pathology.

This *Fmr1* mutant mouse exhibits several phenotypes similar to Fragile X syndrome including increased seizure susceptibility and hyperactivity, as well as deficits in spatial and motor learning [7]–[11]. *Fmr1* KO mice display abnormal sensory gating during prepulse inhibition as well as cortical hyperexcitability [12]–[14]. However, whether *Fmr1* KO mice exhibit differences in somatosensory responsiveness akin to the characteristic tactile hypersensitivity to sensory stimuli in FXS is unknown. In addition, whether Fmr1 KO possess normal tactile-dependent learning is unclear.

The gap crossing (GC) learning paradigm is a prime example of a distance detection/object localization task [15]–[17]. In this task, mice are placed on an elevated starting (‘home’) platform in a light-tight enclosure. Mice rely on their whiskers to explore the dark environment and locate a ‘target’ platform. A computer-controlled robotic system adjusts the distances between the two platforms. At short distances, mice perform the task by detecting the target platform with their whiskers and nose, activating whiskers as well as touch receptors in the skin around the nose. At longer distances mice depend exclusively on their whiskers for tactile information [18]. Successful task acquisition requires intact somatosensory ‘barrel’ cortex. Mice improve their performance on this task with experience; this learning yields a greater percentage of successful crossings of a given distance in successive sessions of trials.

Here we examined the cortical responses to whisker stimulation and the rate of tactile learning for *Fmr1* mutants and wild-type (WT) mice. *Fmr1* KO mice displayed greater cortical responses to whisker stimulation, normal initial performance on the gap cross task, but significantly lower improvement with experience at whisker-dependent distances. Thus, we conclude that disruption of *Fmr1* gene function alters cortical responses to sensory stimuli and perturbs tactile learning.

## Materials and Methods

### Mice

FVB wild-type (FVB.129P2-*Pde6b+ Tyrc-ch*/AntJ; stock# 4828, Jackson Laboratory) and *Fmr1* KO mice (FVB.129P2-*Pde6b*+ *Tyrc-ch Fmr1tm1Cgr*/J: stock# 4624, Jackson Laboratory) were maintained and all experiments conducted according to protocols approved by the Children’s Hospital Los Angeles Institutional Animal Care and Use Committee. Mice were anesthetized by isoflurane inhalation and euthanized by carbon dioxide asphyxiation in accordance with approved protocols. The Children’s Hospital Los Angeles Institutional Animal Care and Use Committee specifically approved this study. Protocol number 264-12.

Mice were weaned at P20, group housed with same-sex littermates (3–5 per cage) and food and water were available ad libitum except in gap cross groups. Mice trained and tested on the gap cross were individually housed at the start of training through completion of the 6 days of testing and moderately food restricted as normal chow was allocated on a daily basis to maintain 90–95% initial body weight. All mice were 12–14 weeks of age at the time of the study.

### The Gap Cross Assay

The gap cross assay was performed with a custom-built robot (D.H. Herman, manuscript in preparation). In brief, the gap cross assay system is a closed-loop robotic environment with motor controlled units and sensing elements. The mouse behaves upon raised platforms driven by independent linear actuators. The platforms are equipped with servo-motor doors and positional sensors. Data acquisition and control algorithms are both executed online for real-time dynamic control and offline for more advanced analysis. Independent linear actuators move the Plexiglass platforms to generate a range of gap-distances from nose (<4.5 cm) to whisker (5–8 cm) distances in increments of 0.5 cm. To monitor the location of the mouse, IR motion sensors are at the back and edge of each platform. Near the edge of each platform are servo-controlled doors that prevent exploratory behavior during repositioning of the platforms. The linear motors, servos, and motion sensors are USB controlled through microcontroller boards (Arduino Mega 2560 and the Quadstepper Motor Driver) that feed to a quad-core CPU.

Motor positions are processed on a quad-core CPU using the Arduino and Matlab programming environments. Platform position, door status (open/closed) and feeders are real-time controlled using the Arduino C-based development environment (ADE). Custom-built feeders delivered a small sugar pellet (BioServ, product #F05684) following a successful cross. Motion sensor data are continuously acquired and pre-processed within ADE and are visualized and stored in real time. Specifically, sensor activity is encoded as behavioral performance metrics including successful and failed crossing events. Successful trials are defined as trials in which the mouse approaches the gap and crosses. Failures are defined as trials in which the mouse approaches the gap and then retreats back. This information is computed in real-time. Behaviors are segmented into interactive events at the gap and the system is structured as a two state machine: exploration and adjustment. During exploration, the motors are disabled and the system continuously acquires behavioral data through the motion sensors. During adjustment the doors close to halting exploration and motors reposition the platforms for the next exploration phase. Transitions between the two states are triggered by behavioral events (i.e. successful/failed gap-crossing).

Mice were handled for 10 minutes a day for one week prior to beginning the task. The day before training began, mice were habituated to the gap cross apparatus. They were placed in the chamber with background white noise (60–65 dB) for 20 minutes in white light, immediately followed by 20 minutes in the dark. A bridge was placed over the gap to prevent exploration of the gap and gap crossing behavior. All training sessions took place in a light-tight enclosure in the presence of background white noise. Food was provided to the mice at least one hour after their final training session.

Each training session lasted for 20 successful trials or a maximum of 20 minutes. The training lasted a total of 6 days, with 2 sessions per day for a total of 12 consecutive sessions. Training sessions were separated by at least 6 hours. All sessions began with a trial at 3.0 cm, the shortest distance tested. The position of the mouse was tracked with motion sensors placed at the back and near the edge of each platform. As a mouse traversed the platform, these sensors recorded its progressive position. A successful trial was identified as any trial in which the mouse successfully crossed the gap between the home and target platforms and activated the motion sensor at the back of the target platform. These trials were rewarded with a 5 mg casein pellet delivered from an automated feeder. A failed attempt was defined as an attempt in which the mouse explored the edge of the home platform and returned to the back of the platform. Following each success or failure, the subsequent gap distance was determined using an adaptive learning algorithm designed to decrease the predictability of the next gap distance.

The learning algorithm incorporates the progressive history of successful crosses during a session. Beginning with the first trial at 3.0 cm, the next gap distance was chosen randomly from a uniform distribution of distances (in 0.5 cm increments) 1.0 cm less than the maximum distance crossed in the session (to a minimum of 3.0 cm) to 1.5 cm greater than the maximum distance crossed in the session (to a maximum distance of 7.0 cm). This process was then repeated iteratively until 20 successful trials or 20 minutes had elapsed, completing the session (D.H.Herman, manuscript in preparation).

### Cranial Windows

Male FVB wild-type (WT) and *Fmr1* KO mice (12–14 weeks of age) were used. Mice were anaesthetized with isoflurane (4% induction, 1%–1.5% maintenance) throughout surgery. Body temperature was maintained with a biofeedback heatplate (Physitemp). A circular region of the skull over barrel cortex was thinned to allow visualization of blood vessels at the brain surface without perturbing the underlying dura. A 3 mm diameter #1 thickness cover glass (Bellco) was placed on the thinned skull, affixed with cyanoacrylate and sealed with dental acrylic. A small aluminum bar with tapped screw holes was embedded into the acrylic to stabilize the animal for subsequent imaging sessions. Animals received buprenorphine (0.1 µg/g body weight) and baytril in water (0.1 mg/ml) post-surgery. Their water was also supplemented with carprofen (0.025 mg/ml) throughout the imaging series. Animals were given at least 2 days to recover before intrinsic signal optical imaging.

### Optical Imaging of Intrinsic Signals

Imaging was performed adapted from the temporally-encoded optical imaging approach developed to study visual cortex [19], [20]. Mice were administered chlorprothixene (1 µg/g body weight) prior to imaging and anesthesia was maintained with isoflurane (4% induction, 0.8% to 1.0% maintenance in pure oxygen) delivered through a custom-built nose cone. To visualize whisker-evoked changes in intrinsic signals in S1 barrel cortex, a single whisker (e.g. C2) contralateral to the cranial window was deflected approximately 15 degrees every 20 s with a 3 Hz sinusoidal pulse train for 3 s using a piezoelectric actuator controlled by a function generator (GW Instek). This was repeated 35 consecutive times per trial.

Green light (530 nm±30 nm) was used to visualize cerebral vascularization and red light (620 nm±20 nm) to image intrinsic signals. The imaging plane was focused ∼200–400 µm below the pial surface. Images were acquired at 10 frames per second at 1024×1024 pixels per image at 12-bit depth with a high-speed camera (Dalsa 1M60). Custom acquisition and analysis software (C++ and Matlab) spatially binned images and the magnitude of the response (ΔR/R) at the stimulus frequency was extracted from a complete time series for each pixel by Fourier analysis [19].

## Results

**Figure 1:**
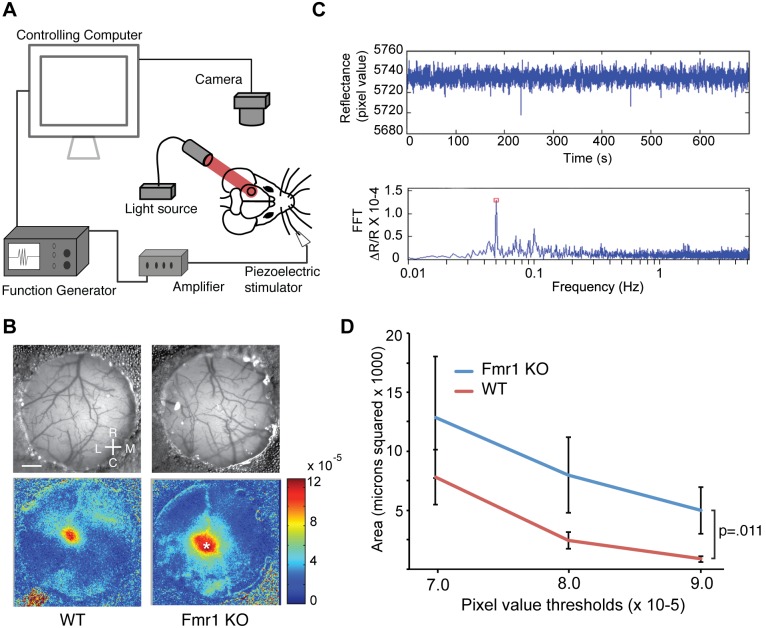
*Fmr1 KO* mice exhibit increased evoked activity in primary somatosensory cortex during whisker stimulation. (A) Schematic showing experimental set up of intrinsic optical imaging over primary somatosensory cortex (black circle) during periodic whisker stimulation. (B) Pictures of the thin skull preparation and example images collected from a wild-type (WT) mouse (left) and *Fmr1* KO mouse (right) mouse. Scale bar = 0.4 mm. Rostral (R), Caudal (C), Lateral (L) and Medial (M) coordinates are shown. (C) Representative examples of data collected during a typical imaging session. Above, a time series of pixel values for the cortical location indicated by the asterisk in the *Fmr1* KO in panel B. Below, a fast-fourier transform (FFT) of the raw trace extracts the magnitude of the change in reflectance (ΔR/R) corresponding to the frequency of whisker stimulation (red square). (D) The number of pixels within the region of response with ΔR/R magnitudes greater than the threshold indicated on the abscissa for WT (n = 10) and *Fmr1* KO (n = 10) mice. The response to whisker stimulation is elevated in *Fmr1* KO mice (WT vs. KO, p = .011; 2-way ANOVA).

To explore the cortical representations of tactile stimuli by *Fmr1* KO and WT mice, we employed optical imaging of intrinsic signals (OIS) to measure cortical responses to whisker stimulation. OIS is a non-invasive measure of tissue reflectance correlated with neural activity [21]–[23]. To measure neural responses to whisker deflection in somatosensory barrel cortex, we adapted a method for measuring intrinsic signals developed to study visual cortex [19] (Fig. 1A, B). We examined the cortical response to stimulation of the C2 whisker for both *Fmr1* mutants and WT mice (Fig. 1C). *Fmr1* KO mice displayed significantly greater size of response (pixels) relative to WT mice across magnitude of response thresholds (Fig. 1D) (Genotype × OIS magnitude threshold: F(1,54) = 6.851, p = .011, KO n = 10, WT n = 10; two-way ANOVA). The size of the region of response in WT mice was approximately the size of barrels observed in slice preparation of mouse cerebral cortex [24]. Thus, Fmr1 mutant mice exhibit abnormally large responses to whisker stimulation in barrel cortex despite a normal cytoarchitecture [25].

**Figure 2:**
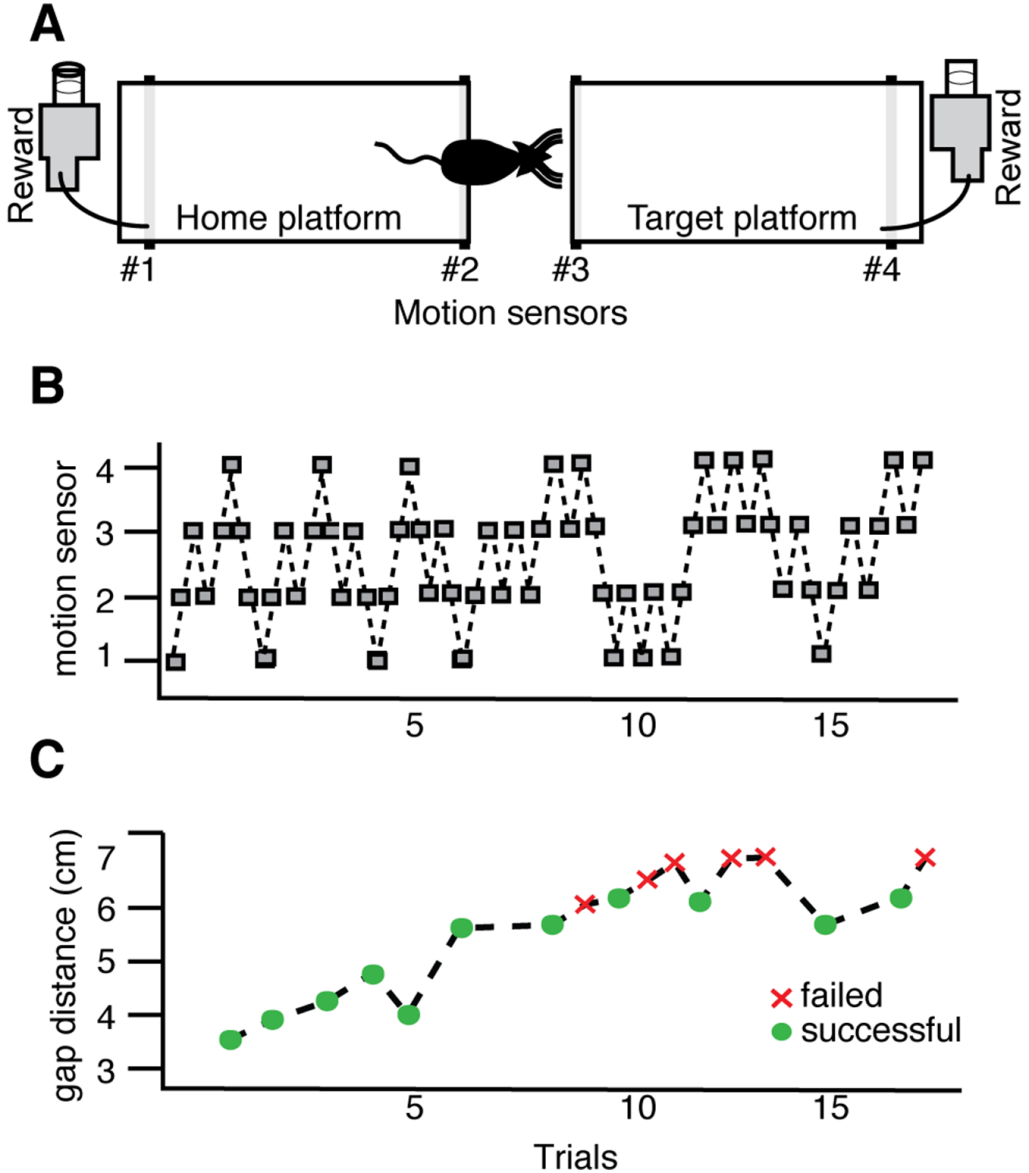
The Gap Cross task is a whisker-dependent sensory learning paradigm. (A) Schematic of the gap cross learning task. Motion sensors positioned at four points along the 2 platforms (labeled #1–4) track the mouse as it moves from the starting platform across a given gap distance to the target platform. (B) Activation of each sensor (grey box) indicates the position of the mouse. (C) Successful crosses are defined as the movement of the mouse from the starting platform to the target platform (green circles). Failures are defined as trials in which the mouse approaches the edge of the home or target platform and returns to the back of the home platform (red crosses).

To determine if *Fmr1* KO mice display associated deficits in whisker-dependent learning, we examined the performance of *Fmr1* KO and WT mice on a validated test of tactile learning, the gap cross task (Figure 2). These were separate cohorts of mice from those examined in OIS experiments (Figure 1). Successful performance on this task requires both tactile stimulation of the whiskers and intact somatosensory cortex [16]–[18]. Following one day of habituation training, all mice were tested on the gap cross at a range of distances spanning 3.0 to 6.0 cm.

**Figure 3:**
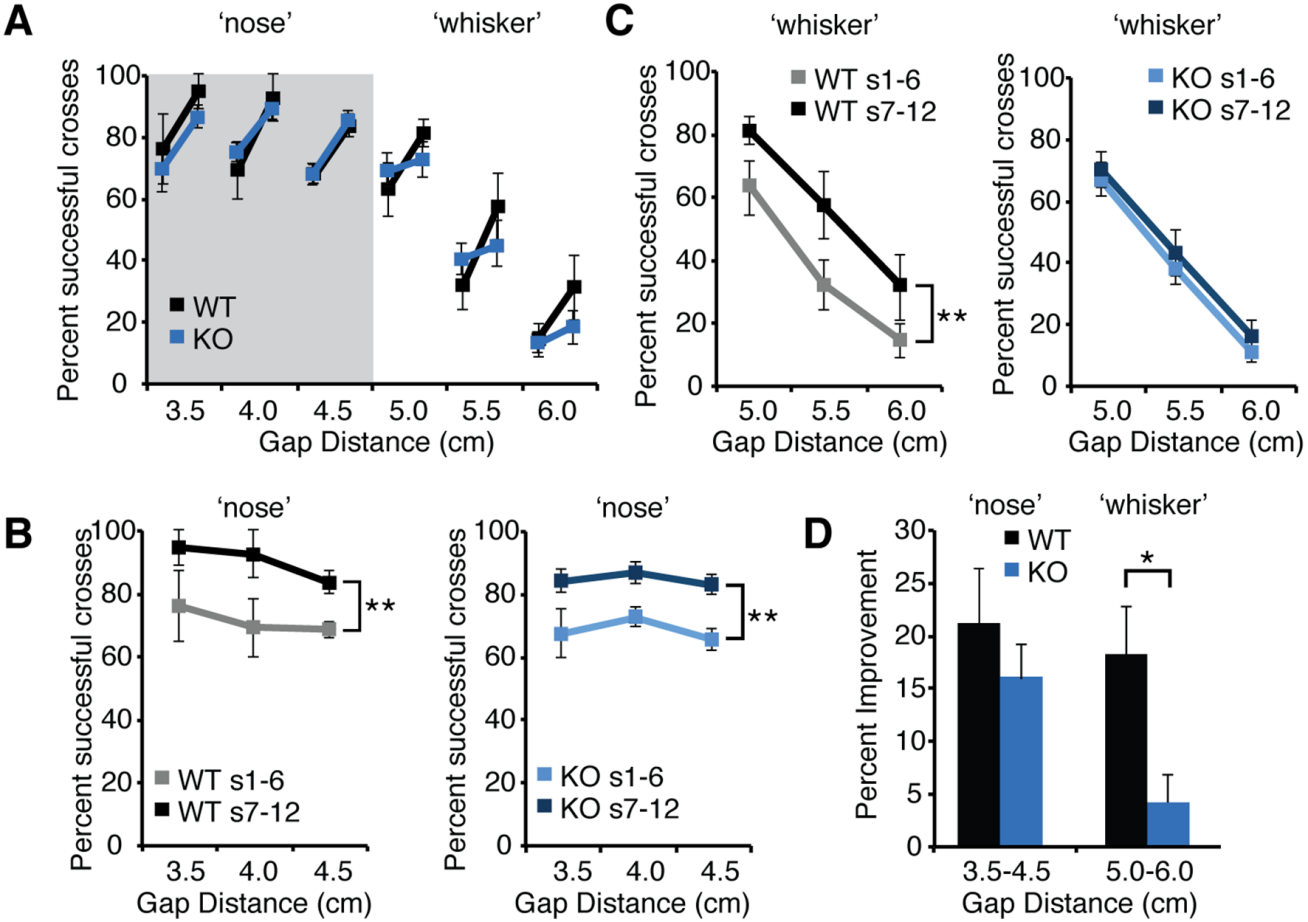
*Fmr1* KO mice display normal learning on the gap cross assay at shorter gap distances but impaired learning at longer whisker-dependent distances. (A) The percent successful crosses averaged across the first six sessions and subsequent six sessions across gap distances ranging from 3.0 cm to 6.0 cm for both wild-type mice (black lines, n = 6) and *Fmr1* KO mice (blue lines, n = 9). For each distance, the line marker on the *left* is the average success rate of the first six sessions and the connected line marker on the *right* is the average success rate of the subsequent six sessions. Error bars represent standard error of the mean. (B) At shorter ‘nose’ distances, both wild-type (WT) and Fmr1 KO mice (KO) improve to a greater percentage of successful crosses between the average of the first six sessions (WT, grey, KO light blue) and the last six sessions (WT, black, KO dark blue). This improvement is statistically significant (WT, p = .007; n = 6; KO, p<.001, n = 9; WT; two-way ANOVA) (C) At whisker-dependent distances, wild-type (WT) improve between early sessions (grey line) and subsequent sessions (black line) despite the lower overall success rate at increasing gap distances. However, KO mice do not display significant improvement between early sessions (light blue line) and later sessions (dark blue line) (WT, p = .002, n = 6, KO, p = .14; n = 9, two-way ANOVA). (D) Average improvement for WT and KO mice at shorter ‘nose’ distances and longer ‘whisker’ distances. WT mice display significantly greater improvement at whisker-dependent distances that KO mice (p = .02, two-tailed t-test).

To assess improvement in performance with experience, we compared the percent of successful crossing between the first six sessions (1–6) and the subsequent six sessions (7–12) at all gap distances tested for WT and *Fmr1* KO mice (Figure 3). Both genotypes displayed a similar high percentage of successful crosses at distances less than 4.5 cm (Fig. 3A, B). At these shorter ‘nose-distances’, mice are able to detect the target platform by touching it with their nose as well as whiskers. WT and *Fmr1* KO mice displayed similar improvement with experience at these ‘nose distances’ (Fig. 3A, B, D). The percent of successful crosses increased significantly in the second half of sessions (sessions 1–6 vs. sessions 7–12) (s1–6 vs. s7–12 X ‘nose’ gap distance, WT, F(1,15) = 9.575, p = .007; n = 6; KO, F(1,24) = 23.41, p<.0001, n = 9; WT; two-way RM-ANOVA) (Fig. 3A, B). Both WT and *Fmr1* KO mice improved an average of more than 15% at these ‘nose’ distances (Fig. 3D).

In contrast to shorter distances, at longer distances (5.0, 5.5, 6.0) mice rely exclusively on information from their whiskers to detect the target platform. Overall, performance declines with increasing gap distance (Fig. 3A). At these longer distances WT mice improved significantly with experience (s1–6 vs. s7–12 X ‘whisker’ gap distance, WT, F(1,15) = 13.60, p = .002, n = 6, two-way RM-ANOVA) (Fig. 3C) and the percentage improvement was similar in magnitude to that at ‘nose distances’ (WT, p>.9; n = 6, two-tailed t-test). Interestingly, *Fmr1* KO mice did not display significant improvement at these whisker-dependent gap distances (s1–6 vs. s7–12 X ‘whisker’ gap distance, KO, F(1,24) = 2.338, p = .14; n = 9, two-way RM-ANOVA) (Fig. 3C). The improvement of *Fmr1* KO mice at ‘whisker’ distances was significantly less than that of WT controls (p = .02, two-tailed t-test) (Fig. 3D). Similarities between the two groups in the total number of trials as well as the number of successful crossings for all distances suggest that these deficits were not due to differences in mobility, exploration or motivation on the task (total number of successful crossings *Fmr1* KO = 195, SEM = +/−7, WT = 183 SEM = +/−9, total number of trials *Fmr1* KO = 366, SEM = +/−8, WT = 369 SEM = +/−24). Thus, *Fmr1* KO mice exhibit a deficit in tactile learning that correlates with abnormal cortical sensory representation of whiskers in barrel cortex.

## Discussion

FXS is characterized by cognitive impairment, anxiety, developmental delay, increased seizure susceptibility, and behavioral hyper-excitability. Altered sensory responses and reduced sensory integration are also symptoms of FXS. In particular, disrupted somatosensory processing, which is often described as tactile defensiveness, is well documented in patients with FXS [5], [26], [27].

*FMR1* KO mice manifest several phenotypes similar to symptoms of FXS [7], [28] These mice have proven to be a useful preclinical model for studying both the molecular functions of FMRP and the morphological and functional disruptions associated with its loss [29]. *Fmr1* KO mice display alterations in synaptic function and plasticity [4], [30]–[36], as well as heightened excitability in barrel cortex that may result from dysfunction within inhibitory circuitry [37]–[39]. In addition, they exhibit increased duration and incidence of network excitation, including increased synchrony of network activity and increased interconnectivity between layer V pyramidal neurons [40]–[42]. Despite both the clinical relevance of sensory dysfunction in Fragile X patients, as well as an increased understanding of the altered network state in *FMR1* KO mice, it remains unclear whether cortical responses to tactile stimulation are aberrant in the somatosensory cortex of *Fmr1* KO mice.

We examined tactile learning by comparing the performance of Fmr1 KO and WT mice on a whisker-dependent sensory learning paradigm, the gap cross task. Fmr1 KO mice displayed initial performance on this task indistinguishable from WT mice and normal learning at shorter ‘nose’ gap distances where sensory information regarding the position of the target platform was presumably more definitive. However, Fmr1 KO mice exhibited significantly less improvement in performance at whisker-dependent distances. Thus, aberrant cortical responses to whisker stimulation we observed in *Fmr1* KO correlate with a deficit in a whisker-dependent and barrel cortex-dependent tactile learning task. However, as OIS is an indirect measure of neural activity, we cannot exclude the possibility that either differences in coupling of neural activity to factors mediating the change in tissue reflectance or different effects of isoflurane on WT and *Fmr1* KO mice could contribute to the differences in cortical responses to whisker stimulation.

However, this perceptual learning task is not exclusively reliant on cerebral cortex or the somatosensory system. Distance detection and object localization integrates motor and sensory activity of both subcortical and cortical circuitry [43], [44]. Thus, whether the exaggerated whisker representations in barrel cortex contribute to the deficits in tactile learning in *Fmr1* KO mice or whether this learning impairment results from aberrant neural circuitry elsewhere in brain is unclear at present. For example, this learning deficit could result from a dysfunction in motor cortex. We considered whether the increased anxiety-like behaviors exhibited by *Fmr1* KO mice might contribute to their deficits in the gap cross assay [9], [45]. However, as the initial performance and improvement of Fmr1 KO mice at shorter ‘nose’ distances was similar to WT mice, as was their initial performance at longer ‘whisker’ distances, we propose that the anxiety-like behaviors of *Fmr1* KO mice are unlikely to be a major contributor to the learning deficit specific to whisker-dependent gap distances we observe. Future studies will be required both to discriminate between these possibilities as well as ascertain if performance by *Fmr1* KO mice on the gap cross assay is improved by administration of mGluR5 antagonists or GABA_B_ agonists reported to rescue some aspects of the phenotype Fmr1 KO mice [46], [47].
